# Dung Beetle Assemblages Attracted to Cow and Horse Dung: The Importance of Mouthpart Traits, Body Size, and Nesting Behavior in the Community Assembly Process

**DOI:** 10.3390/life11090873

**Published:** 2021-08-25

**Authors:** Mattia Tonelli, Victoria C. Giménez Gómez, José R. Verdú, Fernando Casanoves, Mario Zunino

**Affiliations:** 1 Department of Pure and Applied Science (DiSPeA), University of Urbino “Carlo Bo”, 61029 Urbino, Italy; 2 I.U.I CIBIO (Centro Iberoamericano de la Biodiversidad), Universidad de Alicante, San Vicente del Raspeig, 03690 Alicante, Spain; jr.verdu@ua.es; 3 Instituto de Biología Subtropical, Universidad Nacional de Misiones–CONICET, 3370 Puerto Iguazú, Argentina; gimenezgomezvictoria@conicet.gov.ar; 4 CATIE, Centro Agronómico Tropical de Investigación y Enseñanza, 30501 Turrialba, Costa Rica; casanoves@catie.ac.cr; 5 Asti Academic Centre for Advanced Studies, School of Biodiversity, 14100 Asti, Italy; mario.zunino@uniurb.it

**Keywords:** trophic preference, environmental filter, functional diversity, functional traits, niche partitioning, Scarabaeoidea

## Abstract

Dung beetles use excrement for feeding and reproductive purposes. Although they use a range of dung types, there have been several reports of dung beetles showing a preference for certain feces. However, exactly what determines dung preference in dung beetles remains controversial. In the present study, we investigated differences in dung beetle communities attracted to horse or cow dung from a functional diversity standpoint. Specifically, by examining 18 functional traits, we sought to understand if the dung beetle assembly process is mediated by particular traits in different dung types. Species specific dung preferences were recorded for eight species, two of which prefer horse dung and six of which prefer cow dung. Significant differences were found between the functional traits of the mouthparts of the dung beetles attracted to horse dung and those that were attracted to cow dung. Specifically, zygum development and the percentage of the molar area and the conjunctive area differed between horse and cow dung colonizing beetles. We propose that the quantitative differences in the mouthpart traits of the species attracted to horse and cow dung respectively could be related to the differential capacity of the beetles to filtrate and concentrate small particles from the dung. Hence, the dung preference of dung beetles could be related to their ability to exploit a specific dung type, which varies according to their mouthpart traits. Moreover, we found that larger and nester beetles preferred cow dung, whereas smaller and non-nester beetles preferred horse dung. This finding could be related to the tradeoff between fitness and parental investments, and to the suitability of the trophic resource according to the season and species phenology.

## 1. Introduction

Dung beetles (Scarabaeidae: Scarabaeinae, Aphodiinae; Geotrupidae) are distributed in all biogeographic regions and include more than 8000 species [1]. They are mainly coprophagous, a feeding habit which arose from saprophagy in the Cretaceous, leading to a species radiation during mammal diversification in the Cenozoic [2,3]. After the extinction of megafauna, in some places (e.g., Italy, Spain), the feces of domestic livestock became the beetles’ main trophic resource in human-dominated landscapes [4]. In other places (e.g., tropical and subtropical habitats), however, dung beetles became more generalist species associated with the greater diversity and abundance of other types of resource, such as omnivorous dung, vertebrate and invertebrate carrion, fungi, and fruits [5,6]. 

In places where dung is the main source of food, dung beetles use this resource throughout their ontogenetic development, from larval to adult stages [7]. Due to the scarcity of dung in many ecosystems, its scattered occurrence and short existence, dung beetles show an opportunistic and generalized use of a broad range of dung types [8,9,10], and species that are linked exclusively to one kind of dung are very rare [11,12,13,14,15]. Nonetheless, the trophic predilection of dung beetles for certain dung types has often been reported [5,16,17,18,19,20,21,22,23,24], although this preference seems to vary geographically, suggesting that it may not depend on a fixed species trait [25].

Dung is a transient resource with a patchy temporal and spatial distribution [7,8]. Despite its ephemerality, dung is very rich in nutrients, such as minerals, vitamins, carbohydrates, nitrogen, amino acids, and lipids [24,26,27]. Excrement from different species differs markedly in terms of its chemical and physical characteristics [27,28]. Dung from different feeding guilds of vertebrates (i.e., carnivores, omnivores, and herbivores) varies in terms of its composition and proportion of nutrients. Moreover, variations are also found in different species within the same feeding guild [24,27]. For example, the excrement of horses and cows, monogastric and ruminant herbivores, respectively, differs in terms of its nutrient and fiber content, moisture, and volatile organic compounds (VOCs), [24,27,29,30,31]. Different types of feces emit VOCs that are detected by dung beetles through olfactory sensilla on their antennae [32]. Whereas some VOCs are found in all dung types, others seem specific to particular kinds of dung [29,31] and could be used by dung beetles as semio-chemical cues for detecting and selecting their preferred excrement [30,33]. 

Trophic preferences have important consequences for dung beetles because feeding and nesting with different dung types influence their fitness [17,34,35], which in turn can lead to important changes in community structures and compositions over time [36,37,38,39]. What determines the dung preference of dung beetles is, however, a controversial topic. Gittings & Giller [40] found that dung beetle preference is linked to species oviposition behavior. Another factor that could have an effect on preference is the capacity of the beetle to physically make use of the dung. This capacity depends mainly on the moisture and fiber content of the dung; hence, we have the distinction between ‘soft-diet’ and ‘hard-diet’ consumers [41,42,43,44]. The quality of the dung may also have an impact, but findings related to this aspect are conflicting [24,40].

Although several studies have investigated dung beetle biodiversity and species preference for different dung types, to the best of our knowledge, none of these studies has performed a comprehensive analysis using a functional diversity methodology. This approach improves our ecological understanding by focusing on characteristics that define how organisms interact with their physical, chemical, and biological environments [45]. Such an approach could in fact shed light on community assembly processes by examining trait patterns [46,47], where traits are defined as “morphological, biochemical, physiological, structural, phenological, or behavioral characteristics of organisms that influence how they respond to the environment and/or their effects on ecosystem properties” [48]. Thus, the functional diversity approach could provide an additional and complementary source of evidence in addition to taxonomic composition [49,50]. 

Therefore, the purpose of this study was to examine dung beetle assemblages colonizing cow or horse dung in order to determine if differences exist between the two assemblages in terms of species trophic preference and functional traits. We sought to answer two main questions: (i) Are there species which have a preference for cow or horse dung? (ii) Are there some traits linked to a specific dung type? To answer these questions, we focused on multiple (N = 18) species traits related to body morphology, mouthpart morphology, and behavior, seeking to ascertain if the assembly process in different dung types is mediated by these particular traits.

## 2. Materials and Methods

### 2.1. Study Area and Sampling Design to Estimate Trophic Preferences

The study area is located in the sub-mountain pastures of the central Apennine Range (Pesaro-Urbino province, Italy) with an altitudinal range between 500–900 m a.s.l. The climate of the area is temperate. Within the province, we selected five pastures with an extension of between about 20 and 60 hectares (Isola del Piano, Catria, Nerone, Calamello, Pietralata) (see Tonelli et al., [38,51] for further details on the study area and pastures), and within each pasture we selected three sampling sites, separated by at least 500 m to guarantee spatial independence [52,53]. In each sampling site, we placed square with four standardized pitfall traps (CSR model in Lobo et al., [54]) positioned at the corners, separated by about 50 m (four traps × three sampling sites × five pastures = a total of 60 traps). Two of the traps were baited with cow dung (500 cm^3^), while the other two were baited with horse dung (500 cm^3^). The dung was frozen to kill any dung beetles, predators or Diptera larvae that may have altered the results. The data of the two pitfall traps baited with the same type of dung at each sampling site were coupled; hence, there were a total of 30 sampling points (two dung type × three sampling sites × five pastures). Traps were exposed for 48 h every 15 days from June to November 2013 and in May and June 2014. The data of each sampling day were added together to obtain the cumulative abundance and richness of the assemblages. The dung that was used to bait the traps was ivermectin free because this veterinary medical product could alter dung attraction [55]. 

### 2.2. Functional Traits

In total we measured 18 functional traits: 11 morphological (seven associated with the body and four with the mouthpart), and seven behavioral. All traits are associated directly or indirectly with ecological functions. We present a summary table (Table 1) with the potential biological and functional importance of each trait. For further details on the measurement protocols of each trait see Supplementary Materials Files S1 and S2, and Tonelli et al. [56]. 

### 2.3. Data Analysis

#### 2.3.1. Trophic Preferences

Dung beetle preference was analyzed using the Indicator Value Method (IndVal) [57]. This method measures species specificity and fidelity to an ecological status (in our case, fidelity to a dung type). Species with a significant IndVal (*p* < 0.05) were considered to prefer that type of excrement. The analysis was performed using PC-ORD 5 [58]. 

#### 2.3.2. Functional Diversity

Taking into consideration all measured functional traits and all species, we investigated the effect of dung type on functional diversity using single trait indices: *CWM*, *FDvar*, and *FRO*. *CWM* (community weighted mean) represents the expected functional value of one trait in a random community sample. It is the community mean of a trait weighted by the relative abundance of the taxa [59]. *FDvar* (functional divergence) can be defined as the variance of a trait in a community, whose squared residuals are weighted by the abundance of the species [60]. It is constrained between 0–1; high values of functional divergence are related to a high degree of niche differentiation, whereas low values indicate a low level of niche differentiation [61]. *FRO* (functional regularity) is the regularity or evenness of the trait values and abundance in the observed range [62]. It is constrained between 0–1, showing higher values when each species has the same distance from its neighbors and each species is present in the same abundance [62].

The functional diversity indices were calculated using FDiversity software [63]. Dummy variables were created for each category of categorical traits. We used linear mixed models (LMM) to evaluate dung type effect (cow vs. horse) on functional diversity indices. Sampling sites were added as random factors in the LMM procedure in order to avoid any potential effect related to differences in the pastures where the data were collected [64]. When the null hypothesis was rejected, we used Fisher LSD for mean comparison (*p* < 0.05). We evaluated the assumptions of normality and homogeneity of variances using graphical analyses of residuals. Statistical analyses were performed with InfoStat version 2020 [65].

## 3. Results

### 3.1. Trophic Preferences

In total, we collected 156,936 individuals belonging to 58 species. In the horse dung, 90,480 individuals belonging to 50 species were collected (three exclusive species: *Amidorus thermicola* (Sturm, 1800), *Euorodalus paracoenosus* (Balthasar & Hrubant, 1960), *Limarus zenkeri* (Germar, 1813)), whereas in the cow dung, 66,456 individuals belonging to 55 species were collected (eight exclusive species: *Melinopterus stolzi* (Reitter, 1906), *Nialus varians* (Duftschmid, 1805), *Calamosternus mayeri* (Pilleri, 1953), *Bodiloides ictericus* (Laicharting, 1781), *Biralus mahunkaorum* (Ádám, 1983), *Acrossus rufipes* (Linnaeus, 1758), *Agrilinus constans* (Duftschmid, 1805), *Planolinus fasciatus* (Olivier, 1789)) (Table 2). Eight species with a significant trophic preference (IndVal: *p* < 0.05) were identified: two for horse dung and six for cow dung (Table 3).

### 3.2. Functional Diversity

The *CWMs* showed differences in 10 functional traits (*p* < 0.05): four related to body morphology (fresh body mass, hind tibiae length, abdomen length, and wing load), one related to mouthpart morphology (zygum development) and five to ethological traits (nesting pattern, nest type, nest depth, horizontal nest distance, and phenology). Specifically, fresh body mass, hind tibiae length, abdomen length, and wing load showed higher values in cow dung. Regarding mouthpart morphology, we found that the *CWM* of zygum development was higher in horse dung. As regards ethological traits, we found that the *CWM* of nesters (telecoprid and paracoprid with a horizontal and vertical relocation of trophic source) was higher in cow dung, whereas non-nesters that do not relocate dung showed a higher *CWM* in horse dung. Finally, phenology varied significantly between dung type, with a higher *CWM* in species that are active all year or in summer-autumn seasons in the cow dung, while the *CWM* of species actives in autumn, winter and spring was higher in the horse dung. 

The *FRO* of the percentage of molar filtering area was higher in horse dung (*p* < 0.05). The *FDvar* of the percentage of the conjunctive area and the phenology of species actives throughout the year was higher in cow dung (*p* < 0.05) (Table 4).

## 4. Discussion

In the present study we investigated the attractiveness of horse and cow dung to dung beetles using a comprehensive range of functional diversity indices and functional traits. Our investigation showed the presence of dung beetle species with a trophic preference for horse or cow dung. We also found quantitative differences in some mouthpart traits (zygum, conjunctive and molar filtering area) between dung beetles attracted to horse dung and those attracted to cow dung. Furthermore, our results suggest that larger and nester beetles prefer cow dung, whereas smaller and non-nester beetles prefer horse dung. Finally, it was found that phenology could be important to dung beetle trophic selection. 

### 4.1. Trophic Preferences

Our results regarding trophic preferences are consistent with those of Barbero et al. [25] and Dormont et al. [18,29,66]. Previous studies conducted in Europe have found all the species examined in this study in a great variety of dung types [67,68]. Nevertheless, comparing our results with these studies, which explicitly evaluated trophic preference, we found that *Aphodius fimetarius* (Linnaeus, 1758) and *Colobopterus erraticus* (Linnaeus, 1758) were generally more attracted to cow dung [18,66]. For the other species (*Aphodius coniugatus* (Panzer, 1795), *Bodilopsis rufa* (Moll, 1782), *Esymus pusillus* (Herbst, 1789), *Geotrupes spiniger* Marsham, 1802, *Chilothorax conspurcatus* (Linnaeus, 1758) and *Labarrus lividus* (Olivier, 1789)), a trophic preference for cow or horse dung was recorded for the first time. Hence, if this feeding predilection is stable through the species range, it should be investigated in other regions [25]. We also found some species exclusively in horse or cow dung, but their low abundance does not allow us to draw firm conclusions (Table 2).

### 4.2. Mouthparts

**Figure 1:**
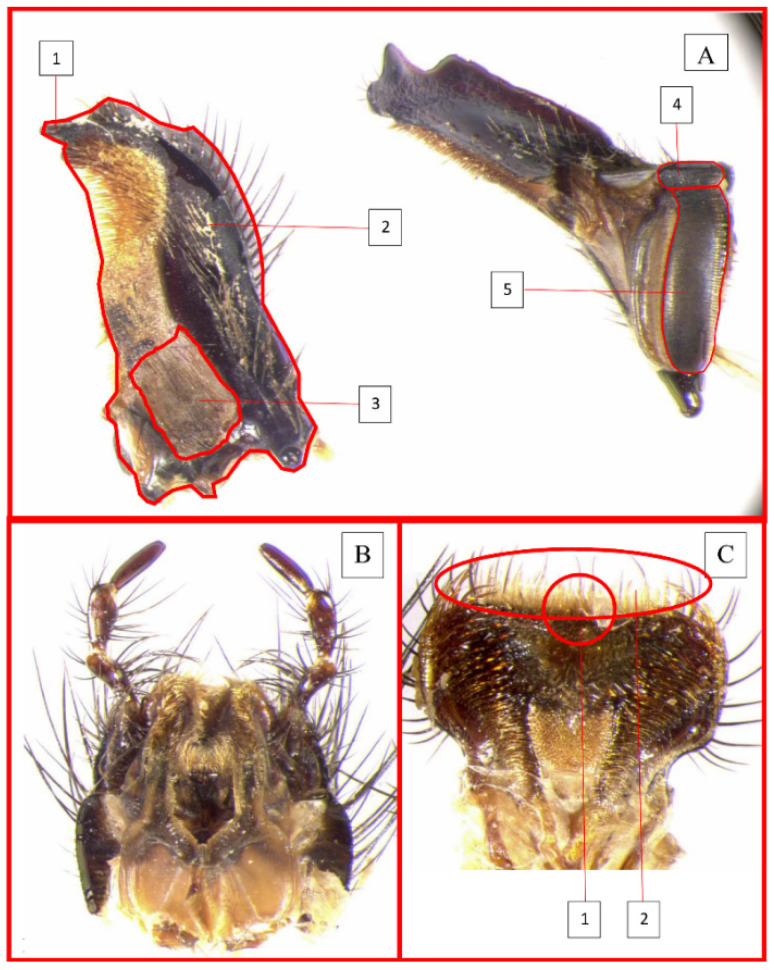
Morphological traits of mouthparts. Example from *Geotrupes spiniger* (Marsham, 1802). (**A**) Mandible and molar area: (1) sclerotized area of the mandible profile (incisor lobo with one tooth); (2) total mandible area; (3) conjunctive; (4) masticator area of the mandibular molar; (5) filter area of the mandibular molar. (**B**) Hypopharynx; (**C**) Epipharynx: (1) Zygum; (2) Acropariae.

Significant differences in the mouthpart traits of the dung beetles attracted to cow dung and those attracted to horse dung were found (Figure 1). Specifically, the zygum, conjunctive and molar area development seems to play an important role during the community assembly process in these two types of dung. The zygum is the proximal part of the epipharynx covered by setae. Its development varies according to the species [69,70]. The conjunctive is a structure whose surface consists of lamellae and it is inserted between the distal and molar lobe of the mandibles [71]. The molar surface consists of tightly packed transverse ridges with numerous pores that connect the molar surface with a system of deeper channels [71,72]. The specific function of these traits is not yet totally clear [71], but their variability seems to be correlated with the type of dung that the beetle feeds on. Indeed, hard and soft feeders show different mouthpart features [41,42,43,44,69,71]. For example, the zygum is more developed in hard dry pellet feeders [69], whereas the development of the conjunctive area is greatly reduced. On the other hand, hard feeders show extensive development of the anterior part of the molar area [71]. In any case, all of these studies draw a distinction between hard feeders and soft feeders based on a qualitative descriptive evaluation of the morphology of their mouthparts. 

The quantitative differences between the mouthpart traits of the species attracted to horse and those attracted to cow dung could thus be related to their differential capacity to filtrate and concentrate small particles from the dung. The greater development of the zygum in dung beetles attracted to horse dung and the reduced variation found in the conjunctive and zygum of these beetles could be associated with the higher overall fiber content and larger fibers found in horse excrement. Horse dung could thus influence the dung beetle community species selection based on these mouthpart features, which may be related to the elimination of coarse fibers. We also found particular mouthpart features associated with cow dung, namely the ratio between the filtering area and the total area of the molar lobes, which showed a diminished level of functional regularity. Hence, we propose that this trait could be linked to the process of concentrating the small particles in cow dung, which has a higher moisture content. 

Horse and cow dung differ in terms of their physical and chemical features [27,29]. One of the most important differences lies in their relative fiber content. Horse dung contains more overall fiber and the fibers themselves tend to be of a larger size [27]. Moreover, horse dung has a lower moisture content [24]. Holter [73] proposed that dung beetles collect dung with their maxillary palps; the large particles are subsequently brushed out by filtration setae on the mouthparts, and the remaining paste is then squeezed by the molar lobes while superfluous liquid is led away from the pharynx through the filtration channels. This process concentrates the remaining small particles, which are then ingested [74]. Hence, there is no evidence that dung beetle mouthparts are involved in the trituration of large particles, and their role seems limited to the elimination of coarse fibers and the concentration of small particles from the aqueous medium. To our knowledge, our study is the first attempt to study the influence of mouthpart traits from a quantitative standpoint.

### 4.3. Body Size and Nesting Behavior

Our results suggest that larger and nester beetles prefer cow dung, whereas smaller and non-nester beetles prefer horse dung. Body size is one of the most important dung beetle traits which is very sensitive to changes in trophic availability and quality [38]. Food quality determines adult dung beetle body size and influences several reproductive outputs [34,35,75]. Larger dung beetles need more dung for nesting, but this necessity is plastic and the amount of dung that is needed may vary according to dung quality and dung type [35,76]. Our results suggest that cow dung could be more suitable for larger dung beetles and can probably maximize their fitness. This result, however, is not in agreement with those reported by other authors who found an optimal investment strategy in dung beetles colonizing horse dung. For example, Hunt & Simmons [34] found that females of the *Onthophagus taurus* need about 20% less dung when provisioning with horse dung, and hence received greater fitness returns per unit of investment and experienced lower provisioning costs in terms of the minimum amount of dung required to produce a surviving offspring than females provisioning with cow dung. Likewise, Moczek [75] observed that almost twice the amount of cow dung was required to yield the body size obtained with half the amount of horse dung. These investigations attribute their findings to the higher quality value of horse dung. However, dung quality can be related to the diet of the animals, showing spatiotemporal variation with seasonal consequences for the trophic preferences and reproductive outputs of dung beetles [77], which could account for the difference in our results.

Another interesting result regards dung beetle nesting behavior. We found that nester species, such as paracoprids and telecoprids, which show relocation behavior [78], prefer cow dung. On the contrary, non-nester species, which lay eggs directly in the dung pat where the entire development takes place, clearly prefer horse dung [79]. This interesting finding is counterintuitive because, generally, non-nester species need to lay their eggs in dung pads characterized by a high water content to avoid desiccation, which could hinder larval development. On the other hand, paracoprid and telecoprid dung beetles bury dung under the soil, where water loss is prevented and coarser and drier dung may not affect larval development [80]. However, our results might also be explained by the phenology of the species that prefer horse or cow dung. Indeed, cow dung was clearly preferred by dung beetles that are active throughout the year or in the summer-autumn period, whereas horse dung was selected by species with a phenological peak during autumn, winter and spring. In the region under study, an average annual precipitation of 1000 mm was recorded with maximum levels in spring and autumn. Hence, non-nester species, such as the *Melinopterus* and *Nimbus* species, can exploit horse dung regardless of its lower moisture content because they are at their phenological peak during wet seasons.

Finally, as a caveat, we must recognize that the collection method can influence the results in trophic preference experiments [81]. Indeed, even though pitfall traps make it possible to conduct large-scale controlled experiments, dung beetles cannot escape from such traps, and this method could reflect the pattern of initial colonization by adult beetles (i.e., findability). On the other hand, collecting with dung pats reflects both findability and preference [18].

## 5. Conclusions

We confirmed the presence of species specific dung beetle trophic preferences for horse or cow dung. Moreover, we found differences in the mouthpart traits of dung beetles between these two excrements. The development of the zygum, as well as the percentage of molar and conjunctive areas, appear to play an important role in the community assembly process and dung choice. This could be related to the difference in the moisture and fiber contents of horse and cow excrement, which selected species based on their ability to filtrate and concentrate small particles. Although dung beetles use volatile organic compounds to discriminate between dung types, their choice could also be related to their capacity to make use of a particular excrement type, which may vary according to their mouthpart morphology. Moreover, larger and nester beetles showed a preference for cow dung, whereas smaller and non-nester beetles preferred horse dung. Larger dung beetles probably maximize their fitness by using cow dung. Generally, non-nester species need a more humid and stable resource (such as ruminant dung), but the presence of these species during the wet season could explain their choice of horse dung. Further studies using more dung types are needed to better understand the role of functional traits in dung beetle trophic preferences and consequent community assembly processes.

## Figures and Tables

**Table 1:** Summary of measured functional traits and their link to ecological functions (See Supplementary Materials Files S1 and S2 for further details). BM = Body Morphology; MM = Mouthpart Morphology; E = Ethological; Quan = Quantitative; Qual = Qualitative.

Trait	Trait Type	Data Type	Functional Link
Fresh body mass	BM	Quan	Resource use
			Metabolic rate
			Thermoregulatory pattern
			Competition
Sphericity	BM	Quan	Resource use
Head area/Total area Ratio	BM	Quan	Resource use
Hind tibiae length	BM	Quan	Resource use
Metamesosternal area	BM	Quan	Dispersal capability
Abdomen length	BM	Quan	Resource use
Wing load	BM	Quan	Dispersal capability
			Foraging strategy
			Habitat use
			Thermoregulatory pattern
Number of teeth in the mandibles profile	MM	Quan	Resource use
Conjunctive/total mandible area ratio	MM	Quan	Resource use
Percentage of filtering/masticator area of mandibular molars	MM	Quan	Resource use
Zygum	MM	Qual	Resource use
Trophic diversity	E	Quan	Resource use
Nest type	E	Qual	Resource use
			Competition
			Habitat use
Nest depth	E	Qual	Resource use
			Competition
			Habitat use
Horizontal nest distance	E	Qual	Resource use
			Competition
			Habitat use
Nesting patterns	E	Qual	Resource use
			Competition
			Habitat use
Daily activity	E	Qual	Resource use
			Competition
			Habitat use
Phenology	E	Qual	Resource use
			Competition
			Habitat use

**Table 2:** Raw data on total dung beetle species and individuals collected in horse and cow dung.

Family	Subfamily	Tribe	Species	Horse	Cow	Total
Scarabaeidae	Aphodiinae	Aphodiini	*Acanthobodilus immundus* (Creutzer, 1799)	6	17	23
Scarabaeidae	Aphodiinae	Aphodiini	*Acrossus luridus* (Fabricius, 1775)	66	163	229
Scarabaeidae	Aphodiinae	Aphodiini	*Acrossus rufipes* (Linnaeus, 1758)	0	4	4
Scarabaeidae	Aphodiinae	Aphodiini	*Agrilinus constans* (Duftschmid, 1805)	0	1	1
Scarabaeidae	Aphodiinae	Aphodiini	*Agrilinus convexus* (Erichson, 1848)	5	18	23
Scarabaeidae	Aphodiinae	Aphodiini	*Amidorus thermicola* (Sturm, 1800)	2	0	2
Scarabaeidae	Aphodiinae	Aphodiini	*Aphodius coniugatus* (Panzer, 1795)	1	10	11
Scarabaeidae	Aphodiinae	Aphodiini	*Aphodius fimetarius* (Linnaeus, 1758)	44	114	158
Scarabaeidae	Aphodiinae	Aphodiini	*Aphodius foetidus* (Herbst, 1783)	4	7	11
Scarabaeidae	Aphodiinae	Aphodiini	*Biralus mahunkaorum* (Ádám, 1983)	0	1	1
Scarabaeidae	Aphodiinae	Aphodiini	*Bodilopsis rufa* (Moll, 1782)	21	808	829
Scarabaeidae	Aphodiinae	Aphodiini	*Bodiloides ictericus* (Laicharting, 1781)	0	11	11
Scarabaeidae	Aphodiinae	Aphodiini	*Calamosternus granarius* (Linnaeus, 1767)	20	3	23
Scarabaeidae	Aphodiinae	Aphodiini	*Calamosternus mayeri* (Pilleri, 1953)	0	1	1
Scarabaeidae	Aphodiinae	Aphodiini	*Chilothorax conspurcatus* (Linnaeus, 1758)	778	3	781
Scarabaeidae	Aphodiinae	Aphodiini	*Chilothorax lineolatus* (Illiger, 1803)	2	1	3
Scarabaeidae	Aphodiinae	Aphodiini	*Chilothorax paykulli* (Bedel, 1907)	48	5	53
Scarabaeidae	Aphodiinae	Aphodiini	*Colobopterus erraticus* (Linnaeus, 1758)	401	2612	3013
Scarabaeidae	Aphodiinae	Aphodiini	*Coprimorphus scrutator* (Herbst, 1789)	45	134	179
Scarabaeidae	Aphodiinae	Aphodiini	*Esymus merdarius* (Fabricius, 1775)	57	50	107
Scarabaeidae	Aphodiinae	Aphodiini	*Esymus pusillus* (Herbst, 1789)	6	31	37
Scarabaeidae	Aphodiinae	Aphodiini	*Euorodalus paracoenosus* (Balthasar & Hrubant, 1960)	2	0	2
Scarabaeidae	Aphodiinae	Aphodiini	*Labarrus lividus* (Olivier, 1789)	663	8	671
Scarabaeidae	Aphodiinae	Aphodiini	*Limarus zenkeri* (Germar, 1813)	2	0	2
Scarabaeidae	Aphodiinae	Aphodiini	*Loraphodius suarius* (Faldermann, 1836)	33	19	52
Scarabaeidae	Aphodiinae	Aphodiini	*Melinopterus consputus* (Creutzer, 1799)	61,128	40,406	101,534
Scarabaeidae	Aphodiinae	Aphodiini	*Melinopterus prodromus* (Brahm, 1790)	6859	531	7390
Scarabaeidae	Aphodiinae	Aphodiini	*Melinopterus reyi* (Reitter, 1892)	12	4	16
Scarabaeidae	Aphodiinae	Aphodiini	*Melinopterus stolzi* (Reitter, 1906)	0	2	2
Scarabaeidae	Aphodiinae	Aphodiini	*Nialus varians* (Duftschmid, 1805)	0	9	9
Scarabaeidae	Aphodiinae	Aphodiini	*Nimbus contaminatus* (Herbst, 1783)	435	371	806
Scarabaeidae	Aphodiinae	Aphodiini	*Nimbus johnsoni* (Baraud, 1976)	12	9	21
Scarabaeidae	Aphodiinae	Aphodiini	*Nimbus obliteratus* (Panzer, 1823)	2175	829	3004
Scarabaeidae	Aphodiinae	Aphodiini	*Otophorus haemorroidalis* (Linnaeus, 1758)	9	63	72
Scarabaeidae	Aphodiinae	Aphodiini	*Phalacronothus biguttatus* (Germar, 1824)	2	4	6
Scarabaeidae	Aphodiinae	Aphodiini	*Planolinus fasciatus* (Olivier, 1789)	0	2	2
Scarabaeidae	Aphodiinae	Aphodiini	*Sigorus porcus* (Fabricius, 1792)	216	179	395
Scarabaeidae	Aphodiinae	Aphodiini	*Teuchestes fossor* (Linnaeus, 1758)	1	11	12
Scarabaeidae	Aphodiinae	Aphodiini	*Trichonotulus scrofa* (Fabricius, 1787)	45	202	247
Scarabaeidae	Scarabaeinae	Onitini	*Bubas bison* (Linnaeus, 1767)	30	76	106
Scarabaeidae	Scarabaeinae	Coprini	*Copris lunaris* (Linnaeus, 1758)	11	12	23
Scarabaeidae	Scarabaeinae	Oniticellini	*Euoniticellus fulvus* (Goeze, 1777)	6129	4130	10,259
Scarabaeidae	Scarabaeinae	Onthophagini	*Caccobius schreberi* (Linnaeus, 1767)	14	16	30
Scarabaeidae	Scarabaeinae	Onthophagini	*Onthophagus coenobita* (Herbst, 1783)	80	126	206
Scarabaeidae	Scarabaeinae	Onthophagini	*Onthophagus fracticornis* (Preyssler, 1790)	3935	6124	10,059
Scarabaeidae	Scarabaeinae	Onthophagini	*Onthophagus grossepunctatus* Reitter, 1905	46	108	154
Scarabaeidae	Scarabaeinae	Onthophagini	*Onthophagus illyricus* (Scopoli, 1763)	1	1	2
Scarabaeidae	Scarabaeinae	Onthophagini	*Onthophagus joannae* Goljan, 1953	297	560	857
Scarabaeidae	Scarabaeinae	Onthophagini	*Onthophagus lemur* (Fabricius, 1781)	194	493	687
Scarabaeidae	Scarabaeinae	Onthophagini	*Onthophagus medius* (Kugelann, 1792)	5273	5601	10,874
Scarabaeidae	Scarabaeinae	Onthophagini	*Onthophagus opacicollis* Reitter, 1892	11	27	38
Scarabaeidae	Scarabaeinae	Onthophagini	*Onthophagus ruficapillus* Brullé, 1832	41	166	207
Scarabaeidae	Scarabaeinae	Onthophagini	*Onthophagus taurus* (Schreber, 1759)	365	506	871
Scarabaeidae	Scarabaeinae	Onthophagini	*Onthophagus verticicornis* (Laicharting, 1781)	564	761	1325
Scarabaeidae	Scarabaeinae	Sisyphini	*Sisyphus schaefferi* (Linnaeus, 1758)	180	781	961
Geotrupidae	Geotrupinae	Geotrupini	*Geotrupes spiniger* Marsham, 1802	91	174	265
Geotrupidae	Geotrupinae	Geotrupini	*Sericotrupes niger* (Marsham, 1802)	115	145	260
Geotrupidae	Geotrupinae	Geotrupini	*Trypocopris vernalis apenninicus* (Mariani, 1958)	3	6	9
			**Total species (S)**	**50**	**55**	**58**
			**Total individuals (N)**	**90,480**	**66,456**	**156,936**

**Table 3:** Dung beetle species that have a trophic preference for cow or horse dung. Numbers represent statistically significant IndVal values (*p* < 0.05).

Indicator Species	Cow	Horse
*Aphodius coniugatus* (Panzer, 1795)	42.4	
*Aphodius fimetarius* (Linnaeus, 1758)	67.3	
*Bodilopsis rufa* (Moll, 1782)	84.5	
*Colobopterus erraticus* (Linnaeus, 1758)	86.7	
*Esymus pusillus* (Herbst, 1789)	61.4	
*Geotrupes spiniger* Marsham, 1802	65.7	
*Chilothorax conspurcatus* (Linnaeus, 1758)		59.8
*Labarrus lividus* (Olivier, 1789)		92.2

**Table 4:** Traits that showed significant differences between dung type (cow and horse) according to the results of *CWM*, *FDvar* and *FRO* indices. The X means a higher significant (*p* < 0.05) value of the index for each trait. More details on these results can be found in the Supplementary Material File S3.

Trait Type	Trait Name	CWM		FDvar		FRO	
		Cow	Horse	Cow	Horse	Cow	Horse
Body Morphology	Fresh body mass	X					
	Hind tibiae length	X					
	Abdomen length	X					
	Wing load	X					
Mouthpart Morphology	Conjunctive/total mandible area ratio			X			
	Percentage of filtering area of mandibular molars						X
	Zygum developed		X				
	Zygum underdeveloped	X					
Ethological	Nest type 0 (non-nester)		X				
	Nest type 3 (Nest composed of a single brood mass located underground in a simple nest)	X					
	Nest type 7 (Nest composed of a single brood ball located underground in a simple nest)	X					
	Nest depth 0 (within excrement)		X				
	Horizontal nest distance 0 (within food source)		X				
	Horizontal nest distance 1 (starting within food source but with a horizontal extension)	X					
	Horizontal nest distance 3 (a great distance out from the food source)	X					
	Nesting patterns 2 (Telecoprid medium-little sized)	X					
	Nesting patterns 5 (Paracoprid with large body size)	X					
	Nesting patterns 8 (Paracoprid with small body size burying dung slowly and at shallow depth without well-developed brood mass)	X					
	Nesting patterns 11 (non-nester)		X				
	Phenology 1 (Autumn, winter and spring)		X				
	Phenology 8 (Summer and autumn)	X					
	Phenology 14 (All year)	X		X			

## Data Availability

The data presented in this study are available in the article.

## Supplementary Materials

The following are available online at https://www.mdpi.com/article/10.3390/life11090873/s1, Supplementary Material S1: Rationale of selected and measured func-tional traits; Supplementary Material S2: Dung beetles average traits values. Supplementary Material S3: Linear Mixed Model regarding the CWM, FDvar and FRO of dung beetle functional traits in cow and horse dung.
