# Supplementary material for: Dung Beetle Assemblages Attracted to Cow and Horse Dung: The Importance of Mouthpart Traits, Body Size, and Nesting Behavior in the Community Assembly Process
Source: Life (Basel). 2021 Aug 25;11(9):873. doi: 10.3390/life11090873 (PMC8472632; doi:10.3390/life11090873)
Supplement: Supplementary file 1 [file life-11-00873-s001.zip › life-1300773 Supplementary material S1 Functional Traits-english done.pdf]

life

MDPI

Article

Dung Beetle Assemblages Attracted to Cow and Horse Dung: The Importance of Mouthpart Traits, Body Size, and Nesting Behavior in the Community Assembly Process

Mattia Tonelli [1,2,*], Victoria C. Giménez Gómez [3], José R. Verdú [2], Fernando Casanoves [4] and Mario Zunino [5]

Citation: Tonelli, M.; Giménez Gómez, V.C.; Verdú, J.R.; Casanoves, F.; Zunino, M. Dung Beetle Assemblages Attracted to Cow and Horse Dung: The Importance of Mouthpart Traits, Body Size, and Nesting Behavior in The Community Assembly Process. *Life* **2021**, *11*, 873. https://doi.org/10.3390/life11090873

Academic Editors: Anita Giglio and Federica Talarico

Received: 30 June 2021
Accepted: 12 August 2021
Published: 25 August 2021

Publisher's Note: MDPI stays neutral with regard to jurisdictional claims in published maps and institutional affiliations.

Copyright: © 2021 by the authors. Submitted for possible open access publication under the terms and conditions of the Creative Commons Attribution (CC BY) license (http://creativecommons.org/licenses/by/4.0/).

Supplementary Material S1: Rationale of selected and measured functional traits

Dung beetle species were characterized by means of 18 functional traits: fresh body mass (quantitative), six morphological body measures (quantitative), four morphological traits of mouthparts (three quantitative and one qualitative) and eight bionomical traits (one quantitative and seven qualitative). Below, we list the functional traits used, their functional significance, the measurement or collection methods used to obtain the data and the number of specimens used to obtain the average value of the trait. The morphological traits were measured by means of the Leica Application Suite software coupled with the Leica M205 C stereo microscope. In order to obtain the average species value for each morphological trait, we randomly selected and measured ten individuals of each species, except for *Trypocopris vernalis apenninicus* Mariani, 1958, of which only four specimens were measured. To avoid any bias due to sexual dimorphism, only females were used (when available). The bionomical traits were obtained from the literature, expert communications and personal observations.

1) Fresh body mass

Fresh body mass is one of the most important functional traits. It is related to the quantity of buried dung (Nervo et al., 2014), metabolic rate (Davis et al., 1999), thermoregulatory pattern (Verdú et al., 2006) and competition (Horgan and Fuentes, 2005). Its relationship with all these parameters defines the functional niche of the species with a strong potential influence on functional diversity. Fresh body mass was measured by weighing live dung beetles with a high-precision scale with 0.1 mg accuracy. We weighed ten specimens for each species (when possible), without separating males and females. Fewer than 10 specimens were weighed for the following species: *Agrilinus convexus* (4), *Caccobius schreberi* (6), *Esymus merdarius* (7), *Esymus pusillus* (8), *Nimbus obliteratus* (5), *Onthophagus coenobita* (5), *Onthophagus grossepunctatus* (4), *Sigorus porcus* (4), *Trichonotulus scrofa* (6) and *Trypocopris vernalis apenninicus* (4).

BODY'S MORPHOLOGICAL TRAITS

1) Sphericity

It is a proxy of dung beetle shape. Shape is an important factor in determining the functional niche of dung beetles by means of resource partitioning. (Hernández et al., 2011). This trait was calculated by the formula of Sneed and Folk (1958): $\sqrt[3]{\left(\frac{b}{a}\right)\left(\frac{c}{b}\right)^3}$ where a = maximum length, b = maximum width, c = maximum depth of profile.

2) Head area/total area ratio

This trait can have a functional implication due to the use of the head during burying behavior or dung disruption.

3) Hind tibiae length

This is an important functional trait because it appears to distinguish the telecoprid Scarabaeinae, which shows a longer hind tibia for modeling and rolling the dung ball

(Inward et al., 2011). This trait was measured as the linear length from femur-tibiae articulation to the distal part of the tibia.

4) Metamesosternal area

It is an indirect trait reflecting the flight capacity of the species because it is linked to the insertion of flight muscles.

5) Abdomen length

This trait is an indirect measure of digestive system length (midgut + hindgut), which may be related to the trophic niche of the species and its digestive capacity (Holter and Scholtz, 2013).

6) Wing load

This trait was measured as the ratio of fresh body mass to total wing area (mg/mm^2). This trait is strongly linked to the dispersal capacity of each species, disentangling the foraging strategy (cruise flight vs. perching) (Peck and Forsyth, 1982; Howden and Nealis, 1975, 1978; Larsen et al., 2008; Silva and Hernández, 2015) and the habitat colonization capacity of the species (Barnes et al., 2014). Moreover, this trait is strongly linked to dung beetle thermoregulatory performances (Merrick and Smith, 2004; Verdú et al., 2004).

MORPHOLOGICAL TRAITS OF MOUTHPARTS[1]

1) Number of teeth in the mandibles profile

When a sclerotized area in the distal lobe of the mandible was found, we further characterized this trait by counting the number of teeth that form this area. This trait may be an indication of hard resource exploitation performance (Figure S1).

2) Conjunctive/total mandible area ratio

Madle (1934) asserts that the conjunctive is a system of salivary channels, while Miller (1961) hypothesized that the "flexible area of the mandible", i.e., the conjunctive, "cushions" the grinding action and permits independent movements of the molar lobes "while the mandibles are in the closed position". However, even though the functional significance of the conjunctive is debated and needs further investigation (Holter, 2004), the presence of conjunctives in all the coprophagous taxa, and their absence in practically all other scarabeids (Nel and Scholtz, 1990; Holter, 2004), makes this trait of great interest, at least from a heuristic standpoint. Indeed, Holter and Scholtz (2011), demonstrated a strong reduction in the conjunctive in pellet feeders compared to wet-dung feeders (Figure S1).

3) The percentage of filtering/masticator area of mandibular molars

We differentiate the area of the mandibular molar area into filtering and masticator areas based on the degree of sclerotization and the directionality of transverse ridges. Although Holter (2000) and Holter et al. (2002) assert that molars of dung beetle do not achieve any grinding, Verdú and Galante, (2004) found a strongly developed masticator area in the mandibular molars of hard-feeding dung beetles (Figure S1).

4) Zygum

The zygum is the central apical part of epipharynx, and it is formed by setae, which were categorized depending on their adaptation toward a hard-feeding diet: 1) underdeveloped, 2) developed and 3) strong prolongation of spatula-shaped epizygum (Verdú and Galante, 2004) (Figure S1).

---

[1] The morphology of mouth parts is an important trait that can divide dung beetles into different trophic niches by enabling them to feed or preventing them from feeding on particular trophic resources. Some studies show that variations in the morphology of denticles of mandibles, molar areas, paraglossae, setae of zygum, setae of acropariae and zygum, may differentiate between "soft-diet consumers" and "hard-diet consumers" (for more details on mouthpart morphology, its ecological significance and dung beetle feeding behavior see: Madle, 1934, Halffter, 1961; Halffter and Matthews, 1966; Bürgis, 1982a, 1982b, 1984a, 1984b; Verdú and Galante, 2004; Dellacasa et al., 2010; Miller, 1961; Hata and Edmonds, 1983; Nel and De Villiers, 1988; Browne and Scholtz, 1999; Nel and Scholtz, 1990; Bai et al., 2015; Holter, 2000, 2004; Holter et al., 2002; Holter and Scholtz, 2011; López-Guerrero and Zunino, 2007).

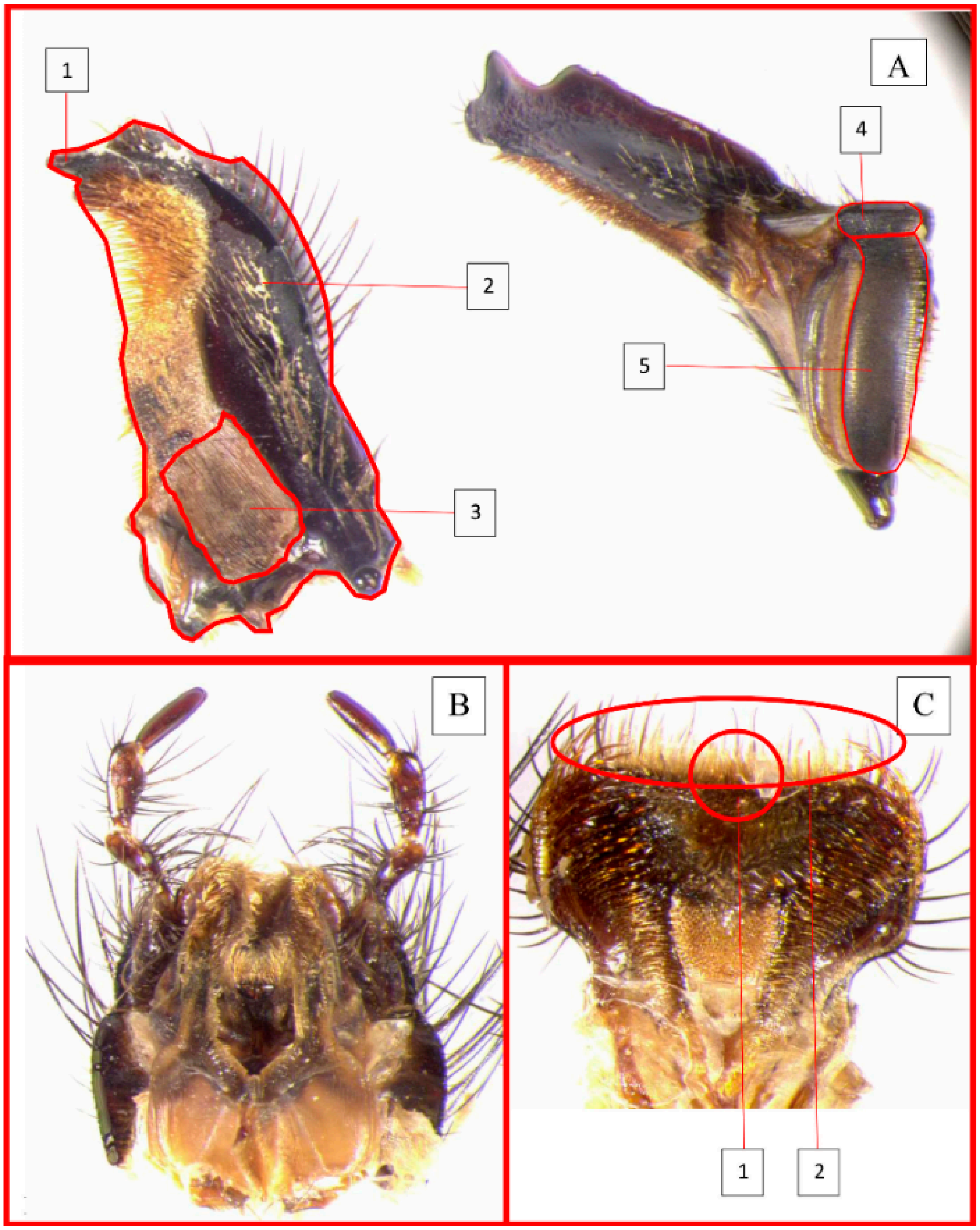

Figure S1. Morphological traits of mouthparts. Example from *Geotrupes spiniger* (Marsham, 1802). (**A**) Mandible and molar area: (1) sclerotized area of the mandible profile (incisor lobo with one tooth); (2) total mandible area; (3) conjunctive; (4) masticator area of the mandibular molar; (5) filter area of the mandibular molar. (**B**) Hypopharynx; (**C**) Epipharynx: (1) Zygum; (2) Acropariae.

BIONOMICAL TRAITS

1) Trophic diversity

Due to the high complexity of the trophic preferences of dung beetles (Barbero et al., 1999; Dormont et al., 2004, 2007, 2010; Errouissi et al., 2004), and their capacity to exploit a range of resources, even resources that are very different from one another (Palestrini and Zunino, 1985), we used an index that represents both the number of aliments that one species may exploit and the qualitative divergence among these aliments. To do this, we developed a hierarchical classification of dung beetle aliments mainly based on their origin and physical conformation (Table S1). For each species, we determined whether or not the trophic resource is used (1,0) based on bibliographical and expert information. Then, we calculated the trophic diversity of each species using an index of taxonomic diversity: the average taxonomic distinctness (Δ+) (Clarke and Warwick, 1998a, 1998b, 2001; Warwick and Clarke, 1995, 1998). This measure takes into account the trophic level to which any two species are related, and it can be thought of as the average length between any two randomly chosen species present in the sample. Hence, each species was characterized by a measure that takes into account the quantity of trophic resources exploited and their divergence in the hierarchical classification.

Table S1. Hierarchical classification of dung beetle trophic resources used to calculate the trophic diversity index.

**Hierarchical level**							**Species (0,1)**
**Food**	**No animal origins**	**Live**	**Fresh resource**			**Fruits**	
						Flowers	
						Mushrooms	
		Death	Not ingested	Decomposed		Decayed plants and mushrooms	
						Decayed fruits	
			Ingested	Ruminants	Mass	Bovine dung	
					Pellets	Ovine, caprine and cervid dung	
				Semi-ruminants	Mass	Camelid and giraffid dung	
					Pellets	Camelid and giraffid dung	
				Monogastrics	Mass	Equine dung	
					Pellets	Rodent and Lagomorph dung	
	Animal origins			Carnivorous and omnivorous		Carnivorous and omnivorous dung	
			Not ingested	Vertebrates	Big size	Big vertebrate carrion	
					Small size	Small vertebrate carrion	
				Invertebrates		Arthropod carrion	
		Live	Fresh resource	Invertebrates		Arthropod predation	

2) Nest type

Due to the high variability of nests (Chapman, 1869, 1870; Halffter and Matthews, 1966; Halffter and Edmonds, 1982; Bornemissza, 1969, 1971; Borghesio and Palestrini, 2002; Brussaard, 1985, 1987; Kirk, 1983; Klemperer, 1978, 1979, 1980, 1981, 1982a. 1982b; Kühne, 1995, 1996; Lumaret, 1975, 1983; Palestrini and Barbero, 1994; Rojewski, 1983;

Romero-Samper and Martín-Piera, 2007; Yoshida and Katakura, 1992; Zunino and Barbero, 1990; Goidanich and Malan, 1964), we differentiated the nester species based on the dung manipulation (masses or balls), nest location (within dung or underground) and nest complexity (simple or compound). The following categories were identified:

0. No-nester
1. Nest composed of a single brood mass located within the excrement;
2. Nest composed of several brood masses located within the excrement;
3. Nest composed of a single brood mass located underground in a simple nest;
4. Nest composed of several brood masses located underground in a simple nest;
5. Nest composed of several isolated brood masses located underground in a compound nest;
6. Nest composed of several brood masses per chamber, located underground in a compound nest;
7. Nest composed of a single brood ball located underground in a simple nest;
8. Nest composed of several isolated brood balls located underground in a simple nest;
9. Nest composed of several brood balls per chamber located underground in a simple nest;
10. Nest composed of several isolated brood balls located underground in a compound nest;
11. Nest composed of several brood balls per chamber located underground in a compound nest.

3) Nest depth

Due to the great variability in nest depth even in species with the same nesting pattern, we differentiated the species based on nest depth into: 0) within excrement, 1) dung-soil interphase, 2) little depth and 3) great depth.

4) Horizontal nest distance

Based on the horizontal distance of the nest relative to the food source, we defined four categories: 0) within food source, 1) starting within food source but with a horizontal extension, 2) a short distance out from the food source and 3) a great distance out from the food source.

5) Nesting patterns

Following the classification of Doube (1990), with some modifications (Tonelli, 2021), we identified the following categories based on nesting behavior and beetle–resource interaction and spatial relationships:

1. Telecoprid 1: large-sized beetles which produce brood balls and show a high interaction with the excrement (i.e., *Scarabaeus*, *Kheper*, *Malagoniella*, *Megathopa* etc.);
2. Telecoprid 2: medium-little sized beetles which produce brood balls and show a high interaction with the excrement (i.e., *Gymnopleurus*, *Sisyphus*, *Canthon* etc.);
3. Telecoprid 3: species not producing brood balls but relocating small-sized dung (rabbit, goat, llama, mara, etc.) without dung molding (i.e., *Eucraniina*, *Thorectes*, *Jekelius*, etc.);
4. Telecoprid 4: species not producing brood balls but relocating small pieces of big dung pats (cow, horse, etc.) without dung molding (i.e., *Bolbites*, *Chalcocopris*, *Trypocopris*, etc.);
5. Paracoprid 1: large body size species burying dung rapidly and at great depth (≥ 50 cm) (i.e., *Copris*, *Bubas*, etc.);
6. Paracoprid 2: large body size species burying dung slowly and at great depth (≥ 50 cm) (i.e., *Onitis*, etc.);
7. Paracoprid 3: small body size species burying dung slowly and at shallow depth (≤ 30 cm) with well-developed brood mass (i.e., *Onthophagus*);
8. Paracoprid 4: small body size species burying dung slowly and at shallow depth (≤ 10 cm) without well-developed brood mass (i.e., *Aphodius*);
9. Endocoprid 1: brood balls developed within dung pat (i.e., *Eurysternus*, *Canthon*, *Oniticellus*, etc.);

10. Cleptocoprid: use of brood masses/balls of other species (i.e., *Aphodius sensu lato*, *Onthophagus sensu lato*, *Caccobius sensu lato*, etc.);
11. No-nest building: eggs are laid within dung pat without brood ball construction (i.e., *Aphodius*, *Trichillum*, *Pedaridium*, etc.).

6) Daily activity

Based on its daily activity pattern we categorized the species as either 1) diurnal or 2) crepuscular/nocturnal.

7) Phenology

Because of the strong seasonality of the dung beetle species, we identified the following phenological patterns based on species activity:

1. Autumn, winter and spring;
2. Winter and spring;
3. Spring;
4. Winter, spring and summer;
5. Spring and summer;
6. Summer;
7. Spring, summer and autumn;
8. Summer and autumn;
9. Summer, autumn and winter;
10. Spring and autumn;
11. Autumn;
12. Autumn and winter;
13. Winter;
14. All year.

References

1. Bai, M.; Li, S.; Lu, Y.; Yang, H.; Tong, Y.; Yang, X. Mandible evolution in the Scarabaeinae (Coleoptera: Scarabaeidae) and adaptations to coprophagous habits. Front. Zoöl. 2015, 12, 1–10, doi:10.1186/s12983-015-0123-z.
2. Barbero, E.; Palestrini, C.; Rolando, A. Dung Beetle Conservation: Effects of Habitat and Resource Selection (Coleoptera: Scarabaeoidea). J. Insect Conserv. 1999, 3, 75–84, doi:10.1023/a:1009609826831.
3. Barnes, A.D.; Emberson, R.M.; Krell, F.-T.; Didham, R.K. The Role of Species Traits in Mediating Functional Recovery during Matrix Restoration. PLOS ONE 2014, 9, e115385, doi:10.1371/journal.pone.0115385.
4. Borghesio, L., Palestrini, C., 2002. Reproductive behavior and larval development in Agrilinus rufus (Moll, 1792) and Oro-mus alpinus (Scopoli, 1763) (Coleoptera: Scarabaeoidea: Aphodiidae). Elytron, 16: 73-79.
5. Bornemissza, G.F., 1969. A new type of brood care observed in the dung beetle Oniticellus cinctus (Scarabae-idae). Pedobiologia, 9: 223-225.
6. Bornemissza, G.F., 1971. A new variant of the paracoprid nesting type in the Australian dung beetle, Onthoph-agus compo-sites. Pedobiologia, 11: 1-10.
7. Browne, J.; Scholtz, C.H. A phylogeny of the families of Scarabaeoidea (Coleoptera). Syst. Èntomol. 1999, 24, 51–84, doi:10.1046/j.1365-3113.1999.00067.x.
8. Brussaard, L., 1985. A pedobiological study of the dung beetle Typhaeus typhoeus (Coleoptera, Geotrupidae). Ph.D. Thesis. Agricultural University, Wageningen, Netherlands. 168 pp.
9. Brussaard, L., 1987. Kleptocopry of Aphodius coenosus in nests of Typhaeus typhoeus and its effect on soil mor-phology. Biology and Fertility of Soils, 3: 117-119.
10. Bürgis, H., 1982a. Gourmets unter den Käfern: Die kotfresser (Coprophaga). I. Hartkotfresser vom Ge-otrupes-Typ. A. Lebensweise und mundwerkzeuge des Stierkäfers. Mikrokosmos, 71: 298-303.
11. Bürgis, H., 1982b. Gourmets unter den Käfern: Die kotfresser (Coprophaga). I. Hartkotfresser vom Ge-otrupes-Typ. B. nahrungsaufnahme der Hartkotfresser. Mikrokosmos, 71: 341-344.
12. Bürgis, H., 1984a. Gourmets unter den Käfern: Die kotfresser (Coprophaga). II. Weichkotfresser vom Aphodi-us-Typ. A. Lebensweise und mundwerkzeuge des Mondhornkäfers. Mikrokosmos, 73: 45-50.
13. Bürgis, H., 1984b. Gourmets unter den Käfern: Die kotfresser (Coprophaga). II. Weichkotfresser vom Aphodi-us-Typ. B.Die nahrungsaufnahme der adulten Weichkotfresser. Mikrokosmos, 73: 368-374.
14. Chapman, T.A., 1869. Aphodius porcus, a cuckoo parasite on Geotrupes stercorarius. Entomologist's monthly Magazine, 5: 273-276.
15. Chapman, T.A., 1870. Further note on the parasitism of Aphodius porcus. Entomologist's monthly Magazine, 6: 230-231.

16. Clarke, K.R.; Warwick, R.M. Quantifying structural redundancy in ecological communities. Oecologia 1998, 113, 278–289, doi:10.1007/s004420050379.
17. Clarke, K.R.; Warwick, R.M.; Pienkowski, M.W.; Watkinson, A.R.; Kerby, G. A taxonomic distinctness index and its statistical properties. J. Appl. Ecol. 1998, 35, 523–531, doi:10.1046/j.1365-2664.1998.3540523.x.
18. Clarke, K.R., Warwick, R.M., 2001. Chanche in marine communities: an approach to statistical analysis and interpretation, 2nd ed. PRIMER-E, Plymouth, UK.
19. Davis, A.L.V.; Chown, S.L.; Scholtz, C.H. Discontinuous Gas-Exchange Cycles in Scarabaeus Dung Beetles (Coleoptera: Scarabaeidae): Mass-Scaling and Temperature Dependence. Physiol. Biochem. Zoöl. 1999, 72, 555–565, doi:10.1086/316698.
20. Dellacasa, G., Dellacasa, M., Mann, D.J., 2010. The morphology of the labrum (epipharynx, ikrioma and aboral surface) of adult Aphodiini (Coleoptera: Scarabaeidae: Aphodiinae), and its implications for systematics. In-secta Mundi, 0132: 1-21.
21. Dormont, L.; Epinat, G.; Lumaret, J.-P. Trophic Preferences Mediated by Olfactory Cues in Dung Beetles Colonizing Cattle and Horse Dung. Environ. Èntomol. 2004, 33, 370–377, doi:10.1603/0046-225x-33.2.370.
22. Dormont, L.; Jay-Robert, P.; Bessière, J.-M.; Rapior, S.; Lumaret, J.-P. Innate olfactory preferences in dung beetles. J. Exp. Biol. 2010, 213, 3177–3186, doi:10.1242/jeb.040964.
23. Dormont, L.; Rapior, S.; McKey, D.B.; Lumaret, J.-P. Influence of dung volatiles on the process of resource selection by coprophagous beetles. Chemoecology 2006, 17, 23–30, doi:10.1007/s00049-006-0355-7.
24. Doube, B.M. A functional classification for analysis of the structure of dung beetle assemblages. Ecol. Èntomol. 1990, 15, 371–383, doi:10.1111/j.1365-2311.1990.tb00820.x.
25. Errouissi, F.; Haloti, S.; Jay-Robert, P.; Janati-Idrissi, A.; Lumaret, J.-P. Effects of the Attractiveness for Dung Beetles of Dung Pat Origin and Size Along a Climatic Gradient. Environ. Èntomol. 2004, 33, 45–53, doi:10.1603/0046-225x-33.1.45.
26. Gittings, T., Giller, P.S., 1998. Resource quality and the colonization and succession of coprophagous dung bee-tles. Ecogra-phy, 21: 581-592.
27. Goidanich A., Malan, C.E., 1964. Sulla nidificazione pedotrofica di alcune specie di Onthophagus Europei e sulla microflora aerobica dell'apparato digerente della larva di Onthophagus taurus Schreber (Coleoptera, Scarabae-idae). Annali della Facoltà di Scienze Agrarie dell'Università degli Studi di Torino, 2: 213-378.
28. Halffter, G., 1961. Monografia de las especies norteamericanas del género Canthon Hoffsg. Ciencia, 20: 225-320.
29. Halffter, G., Edmonds, W.D., 1982. The nesting behavior of dung beetles (Scarabaeinae): An ecological and evolutive ap-proach. Instituto de Ecología Publication 10 México D.F., Man and the Biosphere Program, UNESCO, 176 pp.
30. Halffter, G., Matthews, E.G., 1966. The natural history of dung beetles of the Subfamily Scarabaeinae. Folia En-tomológica Mexicana, (12-14), México, D.F., 313 pp.
31. Hanski, I., 1980. Patterns of beetle succession in droppings. Annales Zoologici Fennici, 17: 17-25.
32. Hata, K.; Edmonds, W. Structure and function of the mandibles of adult dung beetles (Coleoptera : Scarabaeidae). Int. J. Insect Morphol. Embryol. 1983, 12, 1–12, doi:10.1016/0020-7322(83)90031-4.
33. Hernández, M.I.M., Monteiro, L.R., Favila, M.E., 2011. The role of body size and shape in understanding com-petitive interactions within a community of Neotropical dung beetles. Journal of Insect Science, 11(13): 1-14.
34. Holter, P., 2000. Particle feeding in Aphodius dung beetles (Scarabaeidae): old hypotheses and new experi-mental evidence. Functional Ecology, 14: 631-637.
35. Holter, P. Dung feeding in hydrophilid, geotrupid and scarabaeid beetles: Examples of parallel evolution. Eur. J. Èntomol. 2004, 101, 365–372, doi:10.14411/eje.2004.051.
36. Holter, P., Scholtz, C.H., 2011. Re-establishment of biting mouthparts in desert-living dung beetles (Scarabae-idae: Scarabaeinae) feeding on plant litter - old structures reacquired or new ones evolved? Journal of Mor-phology, 272: 1007-1016.
37. Holter, P.; Scholtz, C.H. Elongated hindguts in desert-living dung beetles (Scarabaeidae: Scarabaeinae) feeding on dry dung pellets or plant litter. J. Morphol. 2013, 274, 657–662, doi:10.1002/jmor.20123.
38. Holter, P., Scholtz, C.H., Wardhaugh, K.G., 2002. Dung feeding in adult scarabaeines (tunnelers and endo-coprids): even large dung beetles eat small particles. Ecological Entomology, 27: 169-176.
39. Horgan, F.G., Fuentes, R.C., 2005. Asymmetrical competition between Neotropical dung beetles and its conse-quences for assemblage structure. Ecological Entomology, 30: 182-193.
40. Howden, H.F., Nealis, V.G., 1975. Effects of clearing in a tropical rain forest on the composition of the cop-rophagous Scarab beetle fauna (Coleoptera). Biotropica, 7: 77-83.
41. Howden, H.F., Nealis, V.G., 1978. Observations on height of perching in some tropical dung beetles (Scarabae-idae). Bio-tropica, 10: 43-46.
42. Inward, D.J.G., Davies, R.G., Pergande, C., Denham, A.J., Vogler, A.P., 2011. Local and regional ecological mor-phology of dung beetle assemblages across four biogeographic regions. Journal of Biogeography, 38(9): 1668-1682.
43. Kirk, A.A. The biology of Bubas bison (L.) (Coleoptera: Scarabaeidae) in southern France and its potential for recycling dung in Australia. Bull. Èntomol. Res. 1983, 73, 129–136, doi:10.1017/s0007485300013869.
44. Klemperer, H.G. The repair of larval cells and other larval activities in Geotrupes spiniger Marsham and other species (Coleoptera, Scarabaeidae). Ecol. Èntomol. 1978, 3, 119–131, doi:10.1111/j.1365-2311.1978.tb00910.x.
45. Klemperer, H.G., 1979. An analysis of the nesting behavior of Geotrupes spiniger Marsham (Coleoptera, Scara-baeidae). Ecological Entomology, 4: 133-150.

46. Klemperer, H.G. Kleptoparasitic behaviour of Aphodius rufipes (L.) larvae in nests of Geotrupes spiniger Marsh. (Coleoptera, Scarabaeidae). Ecol. Èntomol. 1980, 5, 143–151, doi:10.1111/j.1365-2311.1980.tb01135.x.
47. Klemperer, H.G. Nest construction and larval behaviour of Bubas bison (L.) and Bubas bubalus (01.) (Coleoptera, Scarabaeidae). Ecol. Èntomol. 1981, 6, 23–33, doi:10.1111/j.1365-2311.1981.tb00969.x.
48. Klemperer, H.G. Normal and atypical nesting behaviour of Copris lunaris (L.): comparison with related species (Coleoptera, Scarabaeidae). Ecol. Èntomol. 1982, 7, 69–83, doi:10.1111/j.1365-2311.1982.tb00645.x.
49. Klemperer, H.G. Parental behaviour in Copris lunaris (Coleoptera, Scarabaeidae): care and defence of brood balls and nest. Ecol. Èntomol. 1982, 7, 155–167, doi:10.1111/j.1365-2311.1982.tb00654.x.
50. Koskela, H., Hanski, I., 1977. Structure and succession in an beetle community inhabiting cow dung. Annales Zoologici Fennici, 14: 204-223.
51. Kühne, R., 1995. Data to the biology of selected Geotrupes species: G. spiniger Marsham, G. vernalis Linné, and G. sterco-rosus Scriba (Coleoptera: Scarabaeidae, Geotrupini). Deutsche Entomologische Zeitscrift, 42(2): 343-367.
52. Kühne, R., 1996. Relations between free-living nematodes and dung-burying Geotrupes spp. (Coleoptera: Ge-otrupini). Fundamental and Applied Nematology, 19(3): 263-271.
53. Larsen, T.H., Lopera, A., Forsyth, A., 2008. Understanding trait-dependent community disassembly: dung bee-tles, density functions, and forest fragmentation. Conservation Biology, 22: 1288-1298.
54. Lobo, J.M., 1992. El relevo microsucesional entre los Scarabaeoidea coprófagos (Col.). Miscellanea Zoologica, Barcelona, 16: 45-59.
55. López-Guerrero, I., Zunino, M., 2007. Consideraciones acerca de la evolución de las piezas bucales en los ont-hophagini (Coleoptera: Scarabaeidae) en relación con diferentes regímenes alimenticios. Interciencia, 32(7): 482-489.
56. Lumaret, J.-P., 1975. Étude des conditions de ponte et de développement larvaire d'Aphodius (Agrilinus) cons-tans Duft. dans la nature et au laboratoire. Vie et Milieu, 25(2) (sér. C): 267-282.
57. Lumaret, J.-P., 1983. La nidification des Trox. Bulletin de la Société Entomologique de France, 88: 594-596.
58. Madle, H., 1934. Zur Kenntnis der Morphologie, Ökologie und Physiologie von Aphodius rufipes LIN. und eini-gen verwandten Arten. Zoologische Jahrbücher (Anatomie und Ontogenie der Tiere), 58: 303-396.
59. Menéndez, R.; Gutiérrez, D. Heterotrophic succession within dung-inhabiting beetle communities in northern Spain. Acta Oecologica 1999, 20, 527–535, doi:10.1016/s1146-609x(00)86620-5.
60. Merrick, M.J., Smith, R.J., 2004. Temperature regulation in burying beetles (Nicrophorus spp.: Coleoptera: Sil-phidae): ef-fects of body size, morphology and environmental temperature. The Journal of Experimental Biolo-gy, 207: 723-733.
61. Miller, A. The Mouth Parts and Digestive Tract of Adult Dung Beetles (Coleoptera: Scarabaeidae), with Reference to the Ingestion of Helminth Eggs. J. Parasitol. 1961, 47, 735, doi:10.2307/3275463.
62. Nel, A., De Villiers, W.M., 1988. Mouthpart structure in adult scarab beetles (Coleoptera: Scarabaeoidea). En-tomologia Generalis, 13(1/2): 095-114.
63. Nel, A., Scholtz, C.H., 1990. Comparative morphology of the mouthparts of adult Scarabaeoidea (Coleoptera). Entomology Memoir Department of Agricultural Development Republic of South Africa, 80: 1-84.
64. Nervo, B.; Tocco, C.; Caprio, E.; Palestrini, C.; Rolando, A. The Effects of Body Mass on Dung Removal Efficiency in Dung Beetles. PLOS ONE 2014, 9, e107699, doi:10.1371/journal.pone.0107699.
65. Palestrini, C., Barbero, E., 1994. The reproductive biology of Aphodius (Coprimorphus) scrutator (Herbst, 1789) (Coleop-tera, Scarabaeidae, Aphodiinae): some experimental data. Bollettino di Zoologia, 61: 60.
66. Palestrini, C., Zunino, M., 1985. Osservazioni sul regime alimentare dell'adulto in alcune specie del genere Thorectes Muls. (Coleoptera, Scarabaeoidea: Geotrupidae). Bollettino del Museo Regionale di Scienze Naturali di Torino, 3(1): 183-190.
67. Peck, S.B., Forsyth, A., 1982. Composition, structure, and competitive behavior in a guild of Ecuadorian rain forest dung beetles (Coleoptera; Scarabaeidae). Canadian Journal of Zoology, 60: 1624-1634.
68. Psarev, A.M., 2001. Succession of the beetle assemblage in cow and horse dung on mountain pastures. Tethys Entomological Research, 3: 125-130.
69. Rojewski, C., 1983. Observations on the nesting behaviour of Aphodius erraticus (L.). Polskie Pismo entomologi-czne, 53: 271-279.
70. Romero-Samper, J., Martín Piera, F., 2007. Comportamiento reproductor y ciclo biológico de Aphodius conjuga-tus (Panzer, 1795) (Coleoptera, Aphodiidae). Boletín Sociedad Entomológica Aragonesa, 41: 189-192.
71. Sabu, T.K.; Vinod, K.V.; Vineesh, P.J. Guild structure, diversity and succession of dung beetles associated with Indian elephant dung in South Western Ghats forests. J. Insect Sci. 2006, 6, 1–12, doi:10.1673/2006_06_17.1.
72. Sabu, T.K., Vinod, K.V., Vineesh, P.J., 2007. Succession of dung beetles (Scarabaeinae: Coleoptera) in the dung pats of gaur, Bos gaurus H. Smith (Artiodactyla: Bovidae), from the moist deciduous forests of Southern West-ern Ghats. Biosystematica, 1(1): 59-69.
73. Da Silva, P.G.; Hernandez, M. Spatial Patterns of Movement of Dung Beetle Species in a Tropical Forest Suggest a New Trap Spacing for Dung Beetle Biodiversity Studies. PLOS ONE 2015, 10, e0126112–e0126112, doi:10.1371/journal.pone.0126112.
74. Sladecek, F.X.J., Hrcek, J., Klimes, P., Konvicka, M., 2013. Interplay of succession and seasonality reflects re-source utiliza-tion in an ephemeral habitat. Acta Oecologica, 46: 17-24.
75. Sneed, E.D.; Folk, R.L. Pebbles in the Lower Colorado River, Texas a Study in Particle Morphogenesis. J. Geol. 1958, 66, 114–150, doi:10.1086/626490.

76. Tonelli, M. Some considerations on the terminology applied to dung beetle functional groups. Ecol. Èntomol. 2021, 46, 772–776, doi:10.1111/een.13017.
77. Verdú, J.R., Arellano, L., Numa, C., 2006. Thermoregulation in endothermic dung beetles (Coleoptera: Scara-baeidae): effect of body size and ecophysiological constraints in flight. Journal of Insect Physiology, 52: 854-860.
78. Verdu, J.R.; Diaz, A.; Galante, E. Thermoregulatory strategies in two closely related sympatric Scarabaeus species (Coleoptera: Scarabaeinae). Physiol. Èntomol. 2004, 29, 32–38, doi:10.1111/j.0307-6962.2004.0359.x.
79. Verdú, J.R.; Galante, E. Behavioural and morphological adaptations for a low-quality resource in semi-arid environments: dung beetles (Coleoptera, Scarabaeoidea) associated with the European rabbit (Oryctolagus cuniculusL.). J. Nat. Hist. 2004, 38, 705–715, doi:10.1080/0022293021000041707.
80. Warwick, R.M., Clarke, K.R., 1995. New 'biodiveristy' measures reveal a decrease in taxonomic distinctness with increasing stress. Marine Ecology and Progress Series, 129: 301-305.
81. Warwick, R.M.; Clarke, K.R.; Pienkowski, M.W.; Watkinson, A.R.; Kerby, G. Taxonomic distinctness and environmental assessment. J. Appl. Ecol. 1998, 35, 532–543, doi:10.1046/j.1365-2664.1998.3540532.x.
82. Yoshida, N., Katakura, H., 1992. Evolution of oviposition habits in Aphodius dung beetles (Coleoptera: Scara-baeidae). Pan-Pacific Entomologist, 68(1): 1-7.
83. Zunino, M., Barbero, E., 1990. Food relocation and the reproductive biology of Aphodius fossor (L.) (Coleoptera Scarabae-idae Aphodiinae). Ethology Ecology & Evolution, 2: 334.
