# Supplementary material for: Dung Beetle Assemblages Attracted to Cow and Horse Dung: The Importance of Mouthpart Traits, Body Size, and Nesting Behavior in the Community Assembly Process
Source: Life (Basel). 2021 Aug 25;11(9):873. doi: 10.3390/life11090873 (PMC8472632; doi:10.3390/life11090873)
Supplement: Supplementary file 1 [file life-11-00873-s001.zip › Supplementary material S3 Linear Mixed Model Results-update.pdf]

life

MDPI

Article

Dung Beetle Assemblages Attracted to Cow and Horse Dung: The Importance of Mouthpart Traits, Body Size, and Nesting Behavior in the Community Assembly Process

Mattia Tonelli [1,2,*], Victoria C. Giménez Gómez [3], José R. Verdú [2], Fernando Casanoves [4] and Mario Zunino [5]

Table S4. Results of the Linear Mixed Model regarding the *CWM*, *FDvar* and *FRO* of dung beetle functional traits in cow and horse dung. The boxes with a slash (/) represent cases in which the absence of variability in the index did not allow us to estimate the model and the pairwise comparison. Significant *P* values are highlighted in bold.

TRAIT TYPE	TRAIT NAME	*CWM*				*FDvar*				*FRO*			
		DUNG TYPE		*F* (1, 24)	*P*	DUNG TYPE		*F* (1, 28)	*P*	DUNG TYPE		*F* (1, 28)	*P*
		Cow	Horse			Cow	Horse			Cow	Horse		
Body Morphology	**Fresh body mass**	**44.612 ± 7.309**	**33.526 ± 7.309**	**12.401**	**0.0017**	**0.85 ± 0.02**	0.83 ± 0.02	0.27	0.6082	0.22 ± 0.02	0.25 ± 0.02	0.94	0.3404
	Sphericity	0.606 ± 0.004	0.607 ± 0.004	1.153	0.2937	2.6 E-03 ± 4.9 E-04	1.7 E-03 ± 4.9 E-04	1.40	0.2464	0.36 ± 0.02	0.36 ± 0.02	0.05	0.8207
	Head area/Total area Ratio	0.097 ± 0.004	0.094 ± 0.004	2.301	0.1424	0.10 ± 0.01	0.08 ± 0.01	2.65	0.1151	0.41 ± 0.02	0.37 ± 0.02	2.75	0.1084
	Hind tibiae length	1.501 ± 0.122	1.365 ± 0.122	17.642	**0.0003**	0.28 ± 0.03	0.21 ± 0.03	3.57	0.0693	0.32 ± 0.02	0.28 ± 0.02	1.98	0.1702
	Metamesosternal area	2.658 ± 0.626	2.313 ± 0.626	2.395	0.1348	0.87 ± 0.01	0.85 ± 0.01	1.88	0.1817	0.26 ± 0.03	0.29 ± 0.03	0.44	0.5128
	Abdomen length	1.707 ± 0.126	1.622 ± 0.126	4.296	**0.0491**	0.23 ± 0.02	0.20 ± 0.02	1.48	0.2344	0.27 ± 0.02	0.27 ± 0.02	0.01	0.9376
	Wing load	0.874 ± 0.158	0.748 ± 0.158	5.101	**0.0333**	0.70 ± 0.05	0.59 ± 0.05	3.05	0.0916	0.38 ± 0.02	0.36 ± 0.02	0.66	0.4221
Mouthpart Morphology	Number of teeth in the mandibles profile	0.019 ± 0.005	0.008 ± 0.005	4.251	0.0502	1.00 ± 0.05	0.93 ± 0.05	1.00	0.3259	0.07 ± 0.01	0.08 ± 0.01	0.01	0.9167
	Conjunctive/total mandible area ratio	0.097 ± 0.002	0.098 ± 0.002	0.539	0.4701	0.05 ± 0.01	0.03 ± 0.01	5.17	**0.0308**	0.34 ± 0.02	0.36 ± 0.02	0.89	0.3527
	Percentage of filtering area of mandibular molars	0.882 ± 0.021	0.869 ± 0.021	3.175	0.0875	0.02 ± 2.9 E-03	0.02 ± 2.9 E-03	1.06	0.3125	0.36 ± 0.02	0.41 ± 0.02	5.00	**0.0335**
	Zygum developed	0.591 ± 0.084	0.741 ± 0.084	9.905	**0.0044**	/	/	/	/	0.04 ± 3.1 E-03	0.05 ± 3.1 E-03	1.56	0.2217
	Zygum underdeveloped	0.409 ± 0.084	0.259 ± 0.084	9.905	**0.0044**	/	/	/	/	0.04 ± 3.1 E-03	0.05 ± 3.1 E-03	1.56	0.2217
Ethological	Trophic diversity	60.529 ± 0.499	60.256 ± 0.499	0.901	0.3520	0.03 ± 0.01	0.03 ± 0.01	0.11	0.7414	0.19 ± 0.01	0.19 ± 0.01	2.1 E-04	0.9885

	Nest type 0 (no-nester)	0.402 ± 0.143	0.533 ± 0.143	4.963	**0.0355**	/	/	/	/	0.04 ± 3.4 E-03	0.05 ± 3.1 E-03	1.56	0.2217
	Nest type 1 (Nest composed of a single brood mass located within the excrement)	8.5 E-05 ± 5.0 E-05	0.000 ± 5.0 E-05	2.581	0.1212	0.13 ± 0.06	0.00 ± 0.06	2.15	0.1534	/	/	/	/
	Nest type 3 (Nest composed of a single brood mass located underground in a simple nest)	0.086 ± 0.024	0.017 ± 0.024	7.626	**0.0109**	/	/	/	/	0.04 ± 3.4 E-03	0.05 ± 3.1 E-03	1.56	0.2217
	Nest type 5 (Nest composed of several isolated brood masses located underground in a compound nest)	0.479 ± 0.133	0.440 ± 0.133	0.518	0.4785	/	/	/	/	0.04 ± 3.4 E-03	0.05 ± 3.1 E-03	1.56	0.2217
	Nest type 7 (Nest composed of a single brood ball located underground in a simple nest)	0.032 ± 0.021	0.010 ± 0.021	4.890	**0.0368**	0.53 ± 0.13	0.40 ± 0.13	0.51	0.4814	0.04 ± 3.4 E-03	0.04 ± 3.9 E-03	0.71	0.4151
	Nest type 9 (Nest composed of several brood balls per chamber located underground in a simple nest)	3.7 E-04 ± 2.0 E-04	2.4 E-04 ± 2.0 E-04	0.464	0.5025	0.40 ± 0.12	0.27 ± 0.12	0.57	0.4560	0.04 ± 2.9 E-03	0.04 ± 3.6 E-03	0.02	0.8979
	Nest depth 0 (within excrement)	0.399 ± 0.142	0.532 ± 0.142	5.035	**0.0343**	/	/	/	/	0.04 ± 3.1 E-03	0.05 ± 3.1 E-03	1.56	0.2217
	Nest depth 1 (dung-soil interphase)	0.008 ± 0.003	0.003 ± 0.003	3.683	0.0669	0.93 ± 0.10	0.67 ± 0.10	3.50	0.0719	0.04 ± 2.8 E-03	0.04 ± 3.4 E-03	0.30	0.5914
	Nest depth 2 (little depth)	0.279 ± 0.090	0.224 ± 0.090	2.167	0.1540	/	/	/	/	0.04 ± 3.1 E-03	0.05 ± 3.1 E-03	1.56	0.2217
	Nest depth 3 (great depth)	0.314 ± 0.083	0.241 ± 0.083	2.849	0.1044	/	/	/	/	0.04 ± 3.1 E-03	0.05 ± 3.1 E-03	1.56	0.2217
	Horizontal nest distance 0 (within food source)	0.956 ± 0.020	0.984 ± 0.020	8.042	**0.0091**	/	/	/	/	0.04 ± 3.1 E-03	0.05 ± 3.1 E-03	1.56	0.2217
	Horizontal nest distance 1 (starting within food source but with a	0.012 ± 0.003	0.006 ± 0.003	4.780	**0.0388**	/	/	/	/	0.04 ± 3.1 E-03	0.05 ± 3.1 E-03	1.56	0.2217

horizontal extension)												
Horizontal nest distance 3 (a great distance out from the food source)	0.032 ± 0.021	0.010 ± 0.021	4.890	**0.0368**	0.53 ± 0.13	0.40 ± 0.13	0.51	0.4814	0.04 ± 3.4 E-03	0.04 ± 3.9 E-03	0.71	0.4151
Nesting patterns 2 (Telecoprid medium-little sized)	0.032 ± 0.021	0.010 ± 0.021	4.890	**0.0368**	0.53 ± 0.13	0.40 ± 0.13	0.51	0.4814	0.04 ± 3.4 E-03	0.04 ± 3.9 E-03	0.71	0.4151
Nesting patterns 5 (Paracoprid with large body size)	0.015 ± 0.005	0.006 ± 0.005	7.488	**0.0115**	/	/	/	/	0.04 ± 3.1 E-03	0.05 ± 3.1 E-03	1.56	0.2217
Nesting patterns 7 (Paracoprid with small body size burying dung slowly and at shallow depth with well-developed brood mass)	0.464 ± 0.134	0.434 ± 0.134	0.326	0.5731	/	/	/	/	0.04 ± 3.1 E-03	0.05 ± 3.1 E-03	1.56	0.2217
Nesting patterns 8 (Paracoprid with small body size burying dung slowly and at shallow depth without well-developed brood mass)	0.086 ± 0.024	0.017 ± 0.024	7.641	**0.018**	/	/	/	/	0.04 ± 3.1 E-03	0.05 ± 3.1 E-03	1.56	0.2217
Nesting patterns 10 (Cleptocoprid)	0.005 ± 0.001	0.005 ± 0.001	0.038	0.8476	/	/	/	/	0.04 ± 3.1 E-03	0.05 ± 3.1 E-03	1.56	0.2217
Nesting patterns 11 (non-nester)	0.397 ± 0.143	0529 ± 0.143	4.991	**0.0351**	/	/	/	/	0.04 ± 3.1 E-03	0.05 ± 3.1 E-03	1.56	0.2217
Daily activity nocturnal	0.016 ± 0.010	0.034 ± 0.010	2.842	0.1048	/	/	/	/	0.04 ± 3.1 E-03	0.05 ± 3.1 E-03	1.56	0.2217
Daily activity diurnal	0.984 ± 0.010	0.966 ± 0.010	2.842	0.1048	/	/	/	/	0.04 ± 3.1 E-03	0.05 ± 3.1 E-03	1.56	0.2217
Phenology 1 (Autumn, winter and spring)	0.333 ± 0.162	0.478 ± 0.162	6.203	**0.0201**	/	/	/	/	0.04 ± 3.1 E-03	0.05 ± 3.1 E-03	1.56	0.2217
Phenology 2 (Winter and spring)	0.003 ± 0.001	0.001 ± 0.001	2.204	0.1506	0.67 ± 0.13	0.60 ± 0.13	0.13	0.7165	0.04 ± 3.8 E-03	0.04 ± 4.0 E-03	0.14	0.7163
Phenology 3 (Spring)	1.4 E-04 ± 6.9 E-05	0.000 ± 6.9 E-05	2.151	0.1555	0.13 ± 0.06	0.00 ± 0.06	2.15	0.1534	/	/	/	/
Phenology 5 (Spring and summer)	0.320 ± 0.118	0.293 ± 0.118	0.648	0.4287	/	/	/	/	0.04 ± 3.1 E-03	0.05 ± 3.1 E-03	1.56	0.2217

	Phenology 7 (Spring, summer and autumn)	0.285 ± 0.103	0.197 ± 0.103	3.648	0.0681	/	/	/	/	0.04 ± 3.1 E-03	0.05 ± 3.1 E-03	1.56	0.2217
	Phenology 8 (Summer and autumn)	0.016 ± 0.004	0.007 ± 0.004	4.363	**0.0475**	/	/	/	/	0.04 ± 3.1 E-03	0.05 ± 3.1 E-03	1.56	0.2217
	Phenology 11 (Autumn)	0.040 ± 0.012	0.024 ± 0.012	1.388	0.2503	/	/	/	/	0.04 ± 3.1 E-03	0.05 ± 3.1 E-03	1.56	0.2217
	Phenology 14 (All year)	0.002 ± 4.4 E-04	4.8 E-04 ± 4.4 E-04	29.792	**<0.0001**	0.93 ± 0.11	0.47 ± 0.11	9.80	**0.0041**	0.04 ± 2.8 E-03	0.04 ± 4.0 E-03	0.21	0.6544
